# Supplementary material for: Physical distancing and emergency medical services utilization after self-harm in Korea during the early COVID-19 pandemic: A nationwide quantitative study
Source: PLoS One. 2023 May 30;18(5):e0286398. doi: 10.1371/journal.pone.0286398 (PMC10228815; doi:10.1371/journal.pone.0286398)
Supplement: S1 Fig — The weighted average of slope coefficients measured as average annual percent change (AAPC) was -1.77 between 2015 and 2012 (blue line). And annual percent change (APC) was -11.98 between 2019 and 2020 (green line). T-statistics were calculated to verify APC and AAPC slopes in a straight linear relationship (null hypothesis). We verified that the difference between two slopes was significantly different from zero at alpha = 0.050 level. The null hypothesis was rejected since p-value was 0.014 in this joinpoint plot. In terms of results, the APC had changed significantly at the end of 2019. (PDF) [file pone.0286398.s001.pdf]

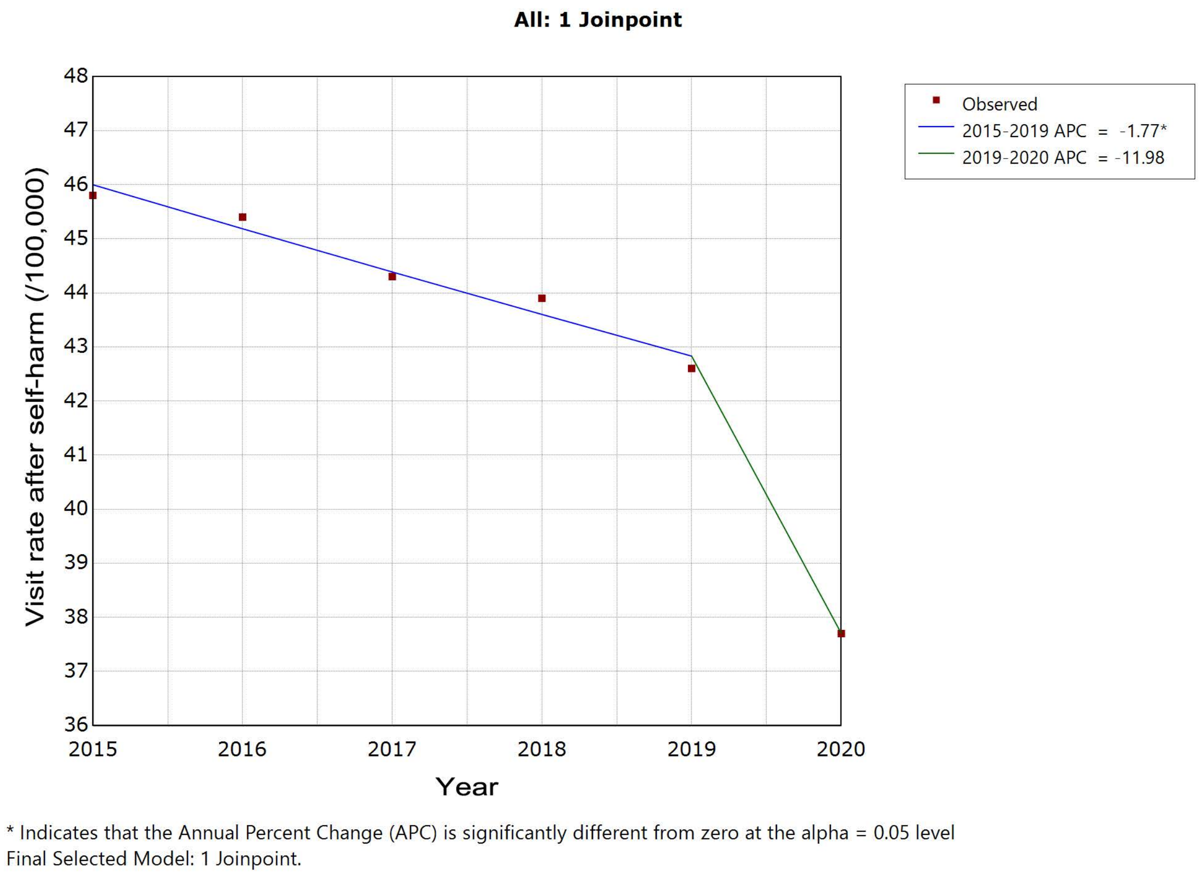

S1 Fig. Graph showing the joinpoint regression model for annual visit rate after self-harm (VRSH) from 2015-2020 with joinpoint at the end of 2019. The weighted average of slope coefficients measured as average annual percent change (AAPC) was -1.77 between 2015 and 2012 (blue line). And annual percent change (APC) was -11.98 between 2019 and 2020 (green line). T-statistics were calculated to verify APC and AAPC slopes in a straight linear relationship (null hypothesis). We verified that the difference between two slopes was significantly different from zero at alpha = 0.050 level. The null hypothesis was rejected since p-value was 0.014 in this joinpoint plot. In terms of results, the APC had changed significantly at the end of 2019.
