# Supplementary material for: Physical distancing and emergency medical services utilization after self-harm in Korea during the early COVID-19 pandemic: A nationwide quantitative study
Source: PLoS One. 2023 May 30;18(5):e0286398. doi: 10.1371/journal.pone.0286398 (PMC10228815; doi:10.1371/journal.pone.0286398)
Supplement: S2 Fig — Cross-correlation function measures the relationship between a time series and a lag version of another time series. This plot showed a maximum correlation coefficient of 0.612 and a lag time of zero weeks. (PDF) [file pone.0286398.s002.pdf]

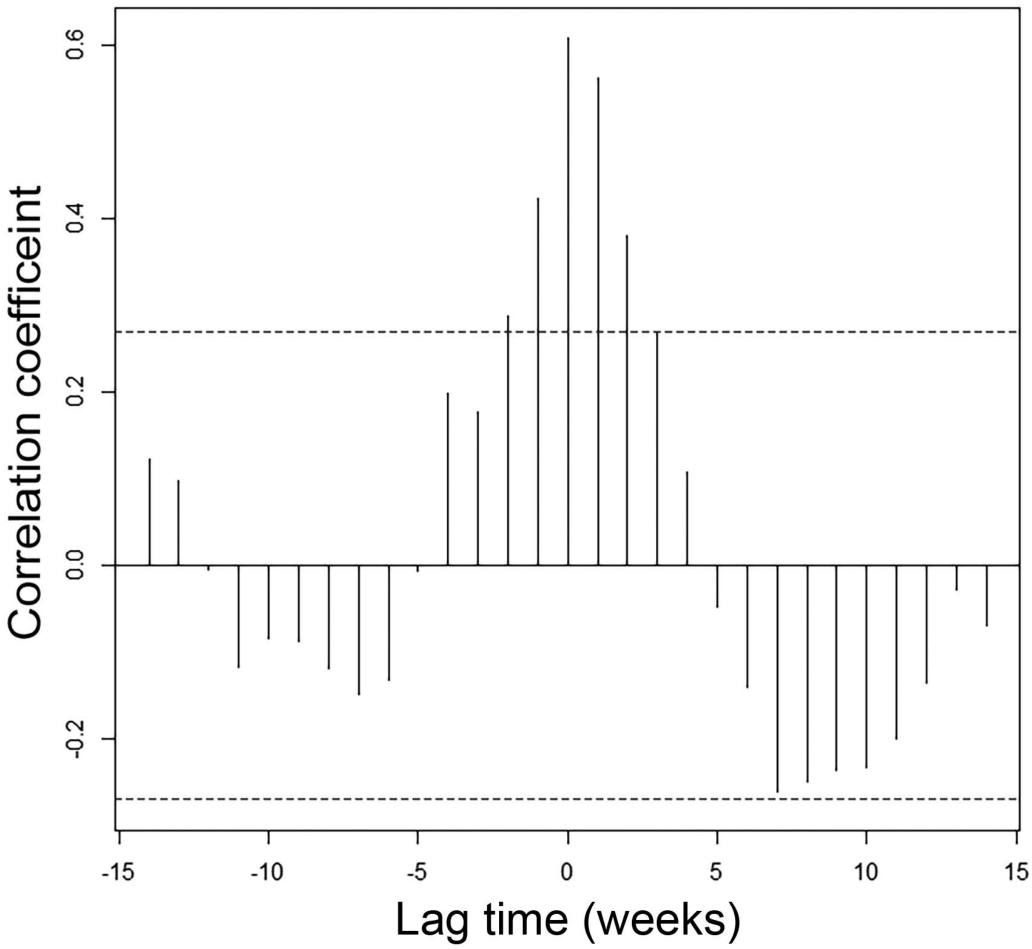

S1 Fig. Cross correlation plot between weekly the mobile phone mobility index and weekly emergency department visit rate after self-harm. Cross correlation function measures the relationship between a time series and a lag version of another time series. This plot shows a correlation coefficient of 0.612 and a lag time of zero weeks.
