# Supplementary material for: Physical distancing and emergency medical services utilization after self-harm in Korea during the early COVID-19 pandemic: A nationwide quantitative study
Source: PLoS One. 2023 May 30;18(5):e0286398. doi: 10.1371/journal.pone.0286398 (PMC10228815; doi:10.1371/journal.pone.0286398)
Supplement: Attachment — Submitted filename: Response_to_peer_reviewers_comments.pdf [file pone.0286398.s003.pdf]

Response to the reviewers' comments and concerns

Dear Dr Zimmerman,

Thank you for giving me the opportunity to submit a revised draft of my manuscript titled "Physical distancing and emergency medical services utilization by self-harm patients in Korea during the early COVID-19 pandemic: A nationwide quantitative study" We appreciate the time and effort that you and the reviewers have dedicated to providing your valuable feedback on my manuscript. We are grateful to the reviewers for their insightful comments on our paper. We have been able to incorporate changes to reflect most of the suggestions provided by the reviewers. We are sending the 'manuscript with track changes' file along with the 'flattened manuscript'.

Here is a point-by-point response to the reviewers' comments and concerns.

Comments from Reviewer 1

General impression: This is a very important manuscript of the secondary effects of Covid-19 pandemic on peoples' mental health and self-harm. I only have a few comments.

Please accept my sincere thanks for reviewing our paper. I have responded to each of the comments below, so please read them and review the manuscript.

Comment 1: You could start the Results section byt one or two general sentence before jumping in to Table 1.

Response: Thank you for your comments and head of the results section has been revised to include a summary of the study findings.

Comment 2: Table 2 Mentality upon arrival to ED: In 2020 the patients seems to be more polarized into two groups alert and unresponsive. Could you discuss why is that.

Response 2: The contents associated with the finding have been added to the upper paragraph of page 16. It would be appreciated if you could review and provide comments.

Comment 3: Discussion: You now start this section telling what the purpose of the study was. This belongs to the end of the introduction and to the Methods. I suggest that you would focus to the main findings of your study in the first paragraph of the discussion.

Response 3: We changed the head of discussion; I would appreciate you rating it.

Comments from Reviewer 2

Please accept my sincere thanks for reviewing our paper. I have responded to each of the comments below, so please read them and review the manuscript.

Introduction

Comment 1: 1) The authors reference other studies that have explored emotional distresses as a result of COVID-19, but they do not refer to any studies from Korea. More description of the situation in Korea during pandemic and the lockdown would be helpful. It could help to get inside to how the population in Korean reacted to the pandemic.

Response: Thank you for your valuable comments. We cited Korean studies that described the effects of pandemic and the lockdown measures for young and elderly adults.

9. Kim HJ, Meeker TJ, Tulloch IK, Mullins J, Park J-H, Bae SH. Pandemic fatigue and anxiety sensitivity as associated factors with posttraumatic stress symptoms among university students in South Korea during the prolonged COVID-19 pandemic. International Journal of Public Health. 2022;67:1604552.
10. Suh BD, Kwon KH. Impacts of the depression among the elderly in the South Korea community in COVID-19 pandemic. Journal of Health Informatics and Statistics. 2021;46(1):54-63.

Comment 2: Line 2 and 4 at the end of the sentences are missing references

Response: Thank you for advice. We added references.

Comment 3: Section 3 after the sentence”…. research has found that peer connections confer a level

of protection against suicide attempts in a variety of conditions.” is missing reference.

Response: Please review the references we cited references emphasize social connectness and friendship network to prevent self-harm and suicide attempts.

Comment 4: In the aim section the authors did not mention (but mention it in the abstract) that they compare the characteristics of self-harm between urban and rural region in the early pandemic (2020).

Response: Based on your point of view, I have revised the introduction section, please reevaluate it.

Methods

Comment 5: Line 4 The authors use incorrect word "mechanism". The correct word would be method of self-harm, which also is used in all theoretical literature and empirical research.

Response: As you pointed out, I have revised it accordingly. Thank you. I also modified the same term in table 4.

Comment 6: **Measurements** Interpretation of incidence rate (IR) is wrong as the denominator has to represent total time at risk (i. e., total person-time). Person-time takes into account the number of people in the group and their time at risk. By calculating weekly incidence rate, the authors should divide by 52. Otherwise, the authors should explain/discuss it.

Response: Your point about incidence is correct. This term cannot be used in this study without investigating the number of occurrences in a risk group or population group. Therefore, we changed the incidence rate to the “ED visit rate after self-harm”. The word "incidence" has been removed. Please re-evaluate the manuscript.

Comment 7: **Statistical analysis** The correct terminology is self-injury method or self-harm method, not injury mechanism. Mechanism refers to something else. Mechanism is why people contemplate or self-harm or what makes people have the this kind of behavior.

Response: We agree with you. All "mechanisms" have been changed to the "self-harm method".

Comment 8: Results Be consistent in choosing urban/rural area or urban/rural region. What is the

proportion of these 2 areas? There is a missing information on percentage of patients in urban and rural areas. This is important information as your study is based on this.

Response: I appreciate you correcting the critical error. Due to the relatively large size of the provinces which we divided, we consolidated them into “region” and revised them.

Comment 9: Results If authors are doing CHI-square analysis, than write Chi values with the P values.

Response: We added chi-square value along with P-value, additionally post-hoc results. Please review again.

Comment 10: Results. Line 4: "…poisoning was the most prevalent cause". Poisoning is a method, not a cause. Cause has a different meaning. Cause of self-harm can be, for example breakup or depression.

Response: We are very sorry for the incorrect terminology and grammatical errors. We modified them.

Comment 11: Results 4.) Correct use of word is poisoning not poisonings. The same problem is with other methods.

Response: We apologize for the grammar error and have corrected them.

Comment 12: Results Line 5 and table 1: In text authors use hanging, in table 1 the authors use choking. Hanging and choking are two different methods. It is either hanging or choking or hanging and choking.

Response: 'Hanging or chocking' is the correct term. So we abbreviated to 'Hanging/chocking'.

Comment 13: Results Line 6: "…who visited the ED through outpatient units was high in rural areas". Don’t the authors mean higher in rural than urban areas?

Response: Thank you for the detailed review. I modified it.

Comment 14: Results Table 1 : check the numbers and percentages again in Table 1
Struck 822 (4.0) 280 (3.3) 1197 (3.9) 822/22421*100=3.67
here should be 3.7

Chocking 893 (3.7) 304 (3.6) 1102 (3.6) 893/22421*100=3.98
here should be 4.0
Beside 304 is wrong number:
1102-893=209 and not 304

Struck (4.0%) and chocking (3.7%) proportion values have been switched. Therefore, we corrected.

Comment 15: Results Text under the table 1: what kind of test are the p-values obtained from? Is it Chi-squared test?
We stated '* Chi-squared test P-values.' in table 1 footnote. Moreover, following the advice of other reviewers, we conducted a Chi-square post-hoc test and included the results. Please review it again.

Comment 16: Results Be consistent in using self-harm or self-injury. In the title the authors use self-harm, then the word should be self-harm.
Thank you. We unified the term.

Comment 17: Results 1 sentence in section under the table: there is something wrong with this sentence "……was higher than in 2019 (+2.35%) than during the same period in 2019".
Changes in the incidence of self-harm during the early stages of the pandemic

Comment 18: Results Section 2: In this sentence: "Compared to the previous five years, the proportion of self-harm patients aged 15 to 34 increased significantly in 2020 (P< 0.046)…". - You cannot merge 2 age groups to one age group when you have 2 age groups with different P-values in the Table 2. 15-24 and 25-34 (p<0.001 and 0.046, respectively).
We have revised. Thank you.

Comment 19: Results ”…. whereas those aged 35 and older decreased significantly (P < 0.050)". Same problem here. P-values are different for each age group. You can write (Table 2).

Response: We have revised. Please review again.

Comment 20: Results Section 2 sentence 4: delete the incidence of stubbing injuries; The authors use incorrect wording of incidence of stubbing : They do not calculate incidence of stubbing. The correct

word is self-harm by stabbing;

Response: It was corrected as "the proportion of stabbings increased to 29.5%,"

Comment 21: Results Again methods, not causes

Response: Thank you so much. That was corrected.

Comment 22: Results Section 2 sentence 5: you cannot merge all methods with one p-value, when the p-values are different for each method

Response: Again, we presented all p-values independently.

Comment 23: Results Section 2 sentence 7: During the pandemic era, 93.5% of patients walked directly to the ER, compared to over 99% in the past (P < 0.001). If you round the number in the past, then you should also round the number during the pandemic. Beside it is almost 100% when rounding off the decimal numbers. You can write (99.7 - 99.8%) instead.

Response: My sincere thanks go out to you for your comments. Updates have been made.

Comment 24: Discussion The authors should discuss that fear of being infected with corona can also lead to reduction in ED visits.

Response: We have discussed on page 16, section **"**The results of this study found that ~ And the implementation of measures that can monitor these changes in real time and take countermeasures is therefore necessary."

Comments from Reviewer 3

Summary: This study aimed to compare the incidence, proportion, demographic and other clinical characteristics of ED visits for self-harm between rural and urban regions in South Korea, during 2020 and 2015-2019. The authors found an overall decrease in ED visits for self-harm in 2020 compared to previous years, which was correlated with mobile mobility measures (a measure of social distancing) suggesting social distancing practices led to a decrease in usage of ED services. They also report ED visits

for self-harm exhibited higher proportions of females and young adults in 2020 compared to previous years. While the aims of this study are important and worth investigating, significant revisions to the methodological and statistical approaches are required to accurately produce and interpret the findings.

Please accept my sincere thanks for reviewing our paper. I have responded to each of the comments below, so please read them and review the manuscript.

Comment 1: Introduction: The introduction states "the study intended to verify that the utilization of emergency care after self-harm increased as physical distancing measures were implemented". Is there more background information to support why the authors made this hypothesis? Most previous literature show decreased use of emergency services due to COVID-19 precautions (including the results of this study!).

Response: One of the major comparisons is between individuals from rural and urban regions. Could the authors present more background on expected differences between these regions?.

Comment 2: Methods ● Measurements: could the authors differentiate between accidental self-harm from intentional self-injury? Intentional-self harm can also occur without suicide intent and this is quite different from accidental self-harm that may not have a significant psychological component. Since one of the goals of this study is to determine how to allocate mental health services to prevent intentional self-harm, it would be better to exclude cases of accidental self-harm from the analyses (or compare the results with and without excluding them).

Response: We defined the self-harm as "The term "self-harm" refers to nonfatal intentional self-injury or self-poisoning, regardless of the apparent motivation or suicidal intent" in Introduction section.

Comment 3: Methods Mobile mobility index - What distance away from the home site would count as one mobile mobility point?

Response: One-mobile phone mobility is recorded when an individual moves from one village

(residence site: staying from midnight to 6 a.m.) to another village (the smallest administrative unit in Korea). The MPMI was calculated by aggregating the total mobile phone mobility and dividing it by the mid-population of each provision. MPMI includes movement statistics for all SK Telecom subscribers aged 15 or older on a weekly basis. This is the data that the National Statistical Office of the Republic of Korea collects and discloses to the public.

Comment 4: Methods ● When comparing urban vs. rural areas, what time period was selected as "early pandemic"? Did this period correspond to significant enforcement of social distancing measures?

Response: Yes, it was. We included the early pandemic period of 2020 in our analysis, and strong distancing regulations were implemented in South Korea during this time period. Measures including a ban on school attendance and a ban on eating in restaurants and cafes were implemented. And the intensity of bans was adjusted to confirmed cases in each province. Therefore, we collected and analyzed the mobile phone mobility index for each province every week.

Comment 5: Methods Two-sample t-tests (comparing rural and urban regions): p-values will need to be adjusted for multiple testing.

Response: We conducted a t-test with all independent variables in the syntax and analyzed results were corrected using the Bonferroni method. We added the related contents to the methods section.

Comment 6: Methods Chi-square test: omnibus chi-square analyses were done for measurements like injury mechanism and indicate overall significant differences in proportions between regions. However, post-hoc chi-square tests may be required to identify individual levels that significantly differ (for example - is choking really significantly less frequent in rural compared to urban areas, or is the difference in poisoning and stabbing driving the omnibus significance?) (1). Of course, adjustments for p-values must also be done to correct for multiple comparisons.

Response: Based on your recommendation, we conducted a chisq post-hoc test for each subgroup of Table 1 after conducting the chisq test on the entire table. It would be greatly appreciated if you could review it.

Comment 7: Methods Correlation-shift-function: could the authors provide more details about the parameters selected using this tool? For example, did the authors perform linear or circular correlations? As well, could the authors provide citations for other papers that have used this software for similar analyses?

Response: We ran the correlation shift app (https://www.originlab.com/FileExchange/details.aspx?fid=466) using Origin Pro (Origin Lab, Northampton, MA), but we suspect the correlation coefficient values were excessively measured. Therefore, we sent queries to OriginLab (You may refer to the bottom of this page for more information about the query: https://www.originlab.com/FileExchange/details.aspx?fid=466), but were not able to receive an answer. Consequently, the authors used R to test the similarity of two curve (MPMI and VRSH) and lag times between the two groups by performing cross-correlation functions in R. Finally, we re-performed statistical analysis R program and have updated all statistical values in the results section. Please review again

Comment 8: Methods What were the effects of selecting other lag-times? How significantly different were the correlations and do these impact confidence in the interpretations?

Response: I have attached a cross correlation (CC) graph for your reference. While the correlation coefficients of VRSH and MPMI will differ from region to region, the CC analysis will generally produce similar graphs. Besides, the lag time is not selected by the authors. Instead, the R program automatically selects the point where maximum height CC appears while moving VRSH and MPMI left and right. The highest CC may be low and the 95% confidence interval may be wide if you choose a different lag time.

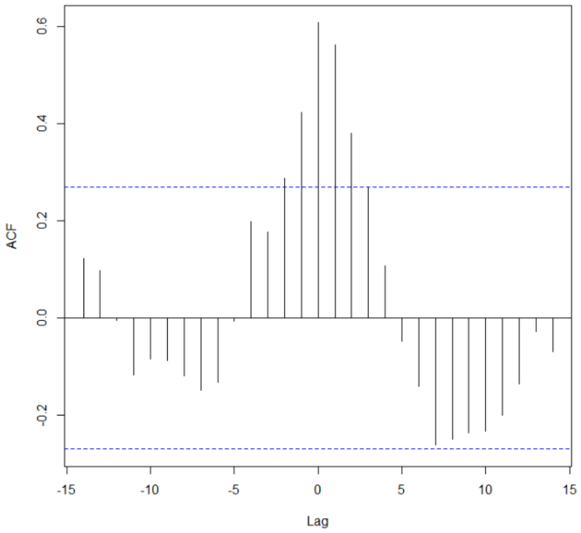

Comment 9: Methods How many urban and rural areas were there?

Response: There are six metropolitan cities classified to urban areas. Rural areas are consisted with 10 provinces. At the beginning of the results, relevant content is described.

Comment 10: Methods Table 1 - general trends in proportions are described but post-hoc statistical tests need to be performed to conclude specific differences such as: In urban areas, the proportion of patients appearing at EDs in an alert mental state was higher than in rural areas (P = 0.046).

Response: Thank you very much for your advice. A chi-square test and a post-hoc test were performed and the results were added to the results section.

Comment 11: Results Table 1 - general trends in proportions are described but post-hoc statistical tests need to be performed to conclude specific differences such as: In urban areas, the proportion of patients appearing at EDs in an alert mental state was higher than in rural areas (P = 0.046).

Response: I completely agree with you. The chi-square coefficient depends on the strength of the relationship and sample size. It had occurred to me that it would be beneficial to add an analysis to measure this trend from the beginning. We analyzed table 1 again and calculated the contingency coefficient (CC), the most employed method to measure the association of x-y in a table. These are also described in the Results section.

Comment 12: Results Here, the p-value is the omnibus chi-square test, not the significance of specific comparisons for "alert mental state" between rural vs urban areas.

Response: I apologize for the error. I conducted a Chisq Post-hoc test and attached the results. As you stated, there is no difference in the level of consciousness between the two regions (P>0.243).

Comment 13: Results SF1 - general trends were described for this figure. However, real statistical tests are required to make conclusions about differences in mobile mobility each week.

Response: Repeated measured ANOVA was performed and the results were added to the results section. S1 Fig. has been changed to Figure 1 and moved into the text. Thank you.

Comment 14: Results This data can actually be used to define specific time-periods of low mobile mobility. It would be interesting to compare intentional self-injury between these time-windows.

Response: There are 16 provinces in Korea. Local governments adjusted the strength of the social distancing measure based on COVID-19 epidemic status. There may were a change in MPMI as a result. If you want to analyze only the MPMI reduction time-windows separately, it should be selected separately for each province. It is also critical to define the reduction criteria. In my opinion, there are too many variables to consider.

The authors tried to find relationship between MPMI as a distancing parameter and VRSH using cross-correlation. Because it was most effective way to find CC and lag time mathematically is by moving the curves of MPMI and VRSH for each local region from one side to the other.

I'd appreciate it if you could take this into consideration.

Comment 15: Results What does the line represent?

Response: It appears that Figure S1 contains a large amount of meaningful data, so I have changed Figure 1. Additionally, the graph lines show the means and error bars present 95% CI for VRSH based (we changed error bars from SEs to 95% confidence intervals). In my opinion, we can realize a clearer picture of the changes because of the lockdown and time period.

Comment 16: Results What is the mobile mobility ratio? What two values are used to make the ratio?

In SF1, the 2020/2019 ratio represents the change in MPMI in 2020 as compared to MPMI in 2019. This graph line shows how MPMI has decreased for each week in 2020 as compared to 2019.

Response: Even in normal years, MPMI varies according to the four seasons and the vacation season. When we first tried the analysis, we tried to show the ratio by dividing MPMI in 2020 by MPMI in 2019. As a result, the ratio has been removed, as the values from the two regions have been presented in graphs for each of 2019 and 2020 (Fig. 1). I would appreciate it if you could review this again.

Comment 17: Results Table 2 - unclear what tests the p-values for each row represent. If these are for t-tests, which years were specifically compared? The results section discusses differences in proportions for some of these results, suggesting chi-square tests were done. If so, why is there a p-value for each row?

Response: It makes sense to use the Chisq test to determine whether the overall distribution varies significantly by group from 2014 to 2020. However, the authors are interested in determining whether there was a significant change in 2020 relative to the previous five years.
Therefore, we compared the proportion (%) in 2020 to the average value of the previous year using a one-sample t-test. Therefore, it is correct that each row has a p-value. And

Comment 18: Results For falling and machine related injuries, were these accidents or purposeful self-harm? If these were accidental, it would be better to remove them from the analyses…
The study does not include unintentional injury cases, as "machine-related" and "falling" self-harm are only included if they are confirmed by triage physicians or nurses as self-harm.
The research method indicates that all subjects included in this study are "intentionally self-injured patients". When collecting NEDIS data, all unintentional injuries or traumas are sent as "unintentional" to the server. Consequently, only "purposeful self-harm" was entered into the research target.

Response: Absolutely correct. Unintentional accidents are coded with a different value. So only self-harm cases are classified as “intentional self-harm” at ED arrival.

Comment 19: Results Table 3 - again, was data from 2020 compared to each of the previous years or an average from the previous years? What was the standard deviation for the 2020 data? Were the units weekly incidence of self-harm per 100 k people?

Response: Incidence is wrong term. Therefore, we changed to correct term "weekly ED visit rate after self-harm per 100,000." in entire manuscript. The SD value could not be obtained for 2020 data because of a single data set. The 2020 values were compared with data from the past five years using a student t-test or a Mann-Whitney U test.

Comment 20: Results T-tests need to be corrected for multiple comparisons for both table 1 and 3. P-value in table 1 is from chisq test. And we did chisq post-hoc test according to your advice and added the results.

Response: We used the one sample test comparing the 2020 value with the previous year's average in table 2 and3. A multiple comparison was not conducted. I would appreciate it if you could take this into consideration and again provide me with your advice.

Comment 21: Results Fig 1, Fig 2, Table 4: The correlation seems extremely high compared to other studies (2). Why is it so high?

Response: We ran the correlation shift app (https://www.originlab.com/fileExchange/details.aspx?fid=466) using Origin Pro (Origin Lab, Northampton, MA), but we suspect the correlation coefficient values were excessively measured. Therefore, we sent queries to OriginLab, but were not able to receive an answer. Consequently, the authors used R to test the similarity of two curve (MPMI and VRSH) and lag times between the two groups by performing cross-correlation functions. Finally, we have updated all statistical values in the results section. Please review again

Comment 22: Results How do other values of time-lags affect the correlations?

Response: When we performed cross correlation with R.
Sample data

```
--------------------------------------------------------------------------------------------------------------------------------
VRSH <-
c(2.09 ,2.03 ,2.04 ,2.25 ,1.99 ,2.02 ,2.04 ,1.85 ,1.64 ,1.68 ,1.78 ,1.84 ,1.84 ,1.93 ,1.91 ,1.92 ,1.97 ,2.13 ,
2.07 ,2.05 ,2.07 ,2.07 ,2.10 ,2.05 ,2.09 ,2.09 ,2.09 ,2.06 ,2.09 ,2.05 ,2.16 ,2.17 ,2.21 ,2.10 ,2.00 ,1.98 ,2.
03 ,2.09 ,2.07 ,2.22 ,2.09 ,2.11 ,2.12 ,2.12 ,2.13 ,2.13 ,2.05 ,2.01 ,1.98 ,1.95 ,1.87 ,1.85 ,1.80 )
MPMI <-
c(0.97 ,0.94 ,0.95 ,1.05 ,0.93 ,0.94 ,0.95 ,0.86 ,0.76 ,0.78 ,0.83 ,0.86 ,0.86 ,0.90 ,0.89 ,0.90 ,0.92 ,0.99 ,
```

0.97 ,0.95 ,0.96 ,0.97 ,0.98 ,0.95 ,0.98 ,0.97 ,0.97 ,0.96 ,0.97 ,0.96 ,1.01 ,1.01 ,1.03 ,0.98 ,0.93 ,0.92 ,0.95 ,0.98 ,0.96 ,1.04 ,0.97 ,0.98 ,0.99 ,0.99 ,0.99 ,0.99 ,0.96 ,0.94 ,0.92 ,0.91 ,0.87 ,0.86 ,0.84)
print(ccf(province, mpmi))

<<Output>>

```
Autocorrelations of series ‘X’, by lag

   -14    -13    -12    -11    -10     -9     -8     -7     -6     -5     -4     -3     -2     -1      0      1 
-0.021 -0.086 -0.097 -0.096 -0.133 -0.147 -0.089 -0.005  0.046  0.108  0.260  0.417  0.561  0.749  0.998  0.755 
     2      3      4      5      6      7      8      9     10     11     12     13     14 
 0.576  0.429  0.261  0.104  0.058  0.008 -0.074 -0.138 -0.126 -0.098 -0.102 -0.090 -0.018 
```

Moving left and right, you can observe a change in CC values as a result of the change in lag time.

Most of the highest correlation occurred around 0-1 week, and the change in correlation according to lag time can be seen in the output data above or the figure below. I would appreciate it if you could refer to them.

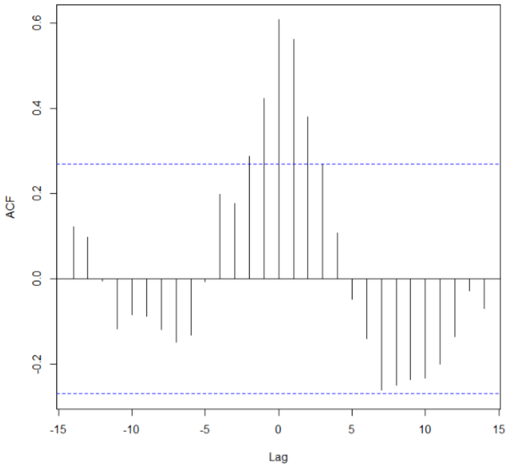

Comment 23: Results How can the average correlations and time-lags be lower in males and females compared to the overall population? Are they statistically lower (you could use a one-sample t-test here).

Response: Thank you for taking the time to review this in detail. In the process of transferring the Origin program output, the CC values and lag time values of the male and female were misrepresented as 0.000 (default). We have updated the original data. It would be greatly appreciated if you could review Table 4 and reevaluate it.

We compared CC value and lag time for two groups (Urban areas (7 metropolitan cities) and rural areas (9 provinces) in Korea) by student T-test.

Comment 24: Discussion The results suggest an increasing trend in self-injury in young people 15-25 over in 2020 compared to previous years. However, there was also a large increase from 2017-2018 in this age group. Is it possible that other factors are contributing to this increase besides the COVID-19 restrictions?

Response: That is a valid point, in my opinion. It is possible that factors such as university entrance examination systems and changes in social welfare may affect self-harm among students aged 15 to 25. There was also a political disruption (transfer of power from the right-wing government to the left-wing government) in 2017. However, the main focus of this study is to compare the 2020 early pandemic period with the previous years. If ED visits after self-harm have increased significantly in the previous years (2017 vs 2018), the one-sample T-test result should show no significance comparing 2020 with previous years. Considering these political factors, it is reasonable to explain this increase as COVID-19 as a result of a significant increase in the 15-25 groups in 2020.

Comment 25: Discussion The results also suggest an increase in incidence and proportion of self-injury in females in 2020 possibly due to COVID-19 restrictions. However, an even larger increase seemed to have occurred between 2017-2018. Since it is unclear if the p-value corresponds to an omnibus test or compared to the average of all years prior to 2020, I'm not sure the authors can make conclusions about incidence.

Response: That is correct, thank you. In 2017 and 2018, the population standardized visit rate for women increased significantly by 19.29% and 13.69% compared to the previous year. There was, however, a reduction in the increase in 2020 to 7.25%. We believe that lockdowns and social distancing will reduce emergency department visit rates after self-harm in 2020, which supports our argument.
I would appreciate it if you could consider this and review it again.

Comment 26: Discussion While the proportion of females seemed to have increased in 2020, the interpretation may be very different depending on if the incidence is different or not.
In Table 2, the proposition of female patients increased significantly in 2020. In addition, it can be seen that the population standardized visibility rate in table 3 also decreases in men, but rather increases in women.

I have concluded that the change in the proportion of females can be explained by the unique increase of population standardized visit rate in the female group.

Response: There are some points on which I agree with you. As a result, however, if population standardized VRSH is to be directly related to changes in female VRSH in the entire population, we must assume that sex ratios in all provinces are the same. Because we can't confirm it like that, I don't think we can assure the crude female visit ratio only with population standard VRSH.
Therefore, we expressed it on page 15 as follows. "In this study, the proportion of females among self-harm patients and the population standardized VRSH among females increased significantly from previous years."
In my opinion, it would be appropriate to present both findings. It would be appreciated if you could review it again after taking this into consideration.

Comment 27: Discussion The authors note that neglecting to get ED treatment after mild self-harm injury is unlikely to occur based on the proportion of alert mental status patients. Can the authors explain this interpretation more? Given the proportion of self-harm ED was lower for older-adults, it's possible that older patients who exhibited intentional self-harm may have perceived the risk of acquiring COVID-19 to be greater than the possible consequences of mild intentional self-harm and chose not to go to the ED. If this was the case, then mental health services should be allocated to older adults as well.

Response: Regarding your concerns, thank you very much. On page 16, we describe the related contents in the second sentence. Patients who self-harm mildly may neglect to visit the ED. Despite that, we attempted to demonstrate that the phenomenon was not significant in this study by citing related studies (reference 56-58). We request that you review this again.

56. Dowell RJ, Ashwood N, Hind J. Musculoskeletal attendances to a minor injury department during a pandemic. Cureus. 2021;13(2). doi: https://doi.org/10.7759/cureus.13143.

57. Pikoulis E, Koliakos N, Papaconstantinou D, Pararas N, Pikoulis A, Fotios-Christos S, et al. The effect of the COVID pandemic lockdown measures on surgical emergencies: experience and lessons learned from a Greek tertiary hospital. World J Emerg Surg. 2021;16(1):1-8. doi: https://doi.org/10.1186/s13017-021-00364-1.

58. Jacob S, Mwagiru D, Thakur I, Moghadam A, Oh T, Hsu J. Impact of societal restrictions and lockdown on trauma admissions during the COVID-19 pandemic: a single-centre cross-sectional observational study. ANZ journal of surgery. 2020;90(11):2227–31. doi: https://doi.org/10.1111/ans.16307.

1. Sharpe D. Chi-Square Test is Statistically Significant: Now What? Pract Assess Res Eval [Internet]. 2019 Nov 25;20(1). Available from: https://scholarworks.umass.edu/pare/vol20/iss1/8
2. Kato H, Takizawa A. Time series cross-correlation between home range and number of infected people during the COVID-19 pandemic in a suburban city. PLOS ONE. 2022 Sep 1;17(9):e0267335.

Response: In the discussion section (page 15), the reference was cited, as well as related findings from this study, including those that differed from the consensus.

Other amendments.

1. We revised the title of manuscript into “Physical distancing and emergency services utilization after self-harm in Korea during the early COVID-19 pandemic.”
2. We Added missing acronyms to Table footnotes.
3. In the study design sub-section (Methods), it is stated that the study is a retrospective before-and-after study.
4. Additionally, the data related to the COVID-19 confirmed cases were from the time we were submitting the manuscript and had a wide gap from the present, so the data is updated as of December 2022.
5. We decided that since a province refers to large-scale land, it should be referred to as a region instead of an area. The word 'area' has been changed to 'region'.
6. We thought S1 figure showing significant contents, so it was moved into the text as Figure 1. In the figure there is a comment about the meaning of ratio and what the line represents, so I redesigned it with a more colorful and revised picture. Please review again.
7. We calculated the proportional values of all tables once again and presented them in the unified vertical direction.
8. In Tables 3, five-year sample values make it difficult to guarantee normality. Thus, the first step was to conduct the Shapiro-Wilk test to verify normality, followed by one-sample t-tests for normal distributions and Wilcox signed run tests for non-normal distributions. The statistical method and

the p-values have also been updated.

9. In table 4, we also chose a parametric (student t-test) or nonparametric test (Mann-Whitney U test), based on the result of the Shapiro-Wilk test, in order to verify the assumption that the sample distribution corresponds to a normal distribution. We updated our statistical methods and results according to this modification. Along with the statistical methods, we added the Shapiro-Wilk W value and updated the p-value.
10. The normality test results have been added to table 3, 4 as well as the type of statistical analysis used to determine the p-value has been updated in the table footnote.
11. Changes in the methods of self-harm are also significant. Therefore, we included a discussion about the changes in self-harm methods that occurred during the pandemic.
12. Harrisco & Co. Korea provided us with English editing services again for grammatical and linguistic errors.
