# Supplementary material for: Physical distancing and emergency medical services utilization after self-harm in Korea during the early COVID-19 pandemic: A nationwide quantitative study
Source: PLoS One. 2023 May 30;18(5):e0286398. doi: 10.1371/journal.pone.0286398 (PMC10228815; doi:10.1371/journal.pone.0286398)
Supplement: Attachment — Submitted filename: Response_to_peer_reviewers_comments.pdf [file pone.0286398.s004.pdf]

Response to the reviewers' comments and concerns

Dear Dr Ooi,

Thank you for giving me the opportunity to submit a revised draft of my manuscript titled "Physical distancing and emergency medical services utilization by self-harm patients in Korea during the early COVID-19 pandemic: A nationwide quantitative study" We appreciate the time and effort that you and the reviewers have dedicated to providing your valuable feedback on my manuscript.

Applying a new statistical method are the most significant changes in revision version 2 that we are submitting. In Table 2 and 3, a one sample t-test was used to compare 2020 values with previous year's values. However, a reviewer pointed out that in this case, continuous upward or downward trends could appear to be significantly changing without being affected by COVID-19. Therefore, the authors were consulted by a statistical specialist. An analysis was conducted using the joinpoint regression program that can analyze these trends, and Tables 2 and 3 were revised.
We revised the results for minor changes, but there have been no significant changes in the existing results in revision version 1.
It would be appreciated if you could review it again considering this.

And, we have been trying to incorporate changes to reflect most of the suggestions provided by the reviewers. We are sending the 'manuscript with track changes' file along with the 'flattened manuscript'.

Here is a point-by-point response to the reviewers' comments and concerns.

Comments from Reviewer 2

Thank you for the revised article. When reading it again, I feel that the article needs major language quality control. There are missing words in the text and the authors mix up past tense with present tense. Please find some (minor) comments below referring to this.

Please accept my sincere thanks for reviewing our paper. I have responded to each of the comments below, so please read them and review the manuscript.

Comment 1: -The authors still use "area" and "region" and "province" in the article. Be consistent in

using one of these words.

Response: The three terms have been unified into "region". Your comment is greatly appreciated.

Comment 2: RESULTS
-the first 6 lines in RESULTS belongs to the Methods

Response 2: We moved the contents to the Methods.

Comment 3: line 6: What time period /year are you referring to in this sentence? "A total of 32,778,938 people lived in the urban regions, and 14,736,844 people lived in the eight rural provinces".

Response 3: The numbers were measured at the mid of 2020 (1st July) and we mentioned that in the methods section.

Comment 4: You wrote "eight rural provinces" but you didn't provide information on how many urban regions there were. You can either delete the 8 or write also number of urban areas.

Response 4: In the second paragraph of the section titled "Study design and data collection", we have included information about urban and rural regions. Please review the revised version.

Comment 5: line 9: Please rephrase this sentence. The wording is imprecise: "In the chi-square post-hoc test for age-specific distribution, the proportion in the 15 – 24 and 25 – 34–year–old groups in the rural area, and the proportion in the over–45–year–old group in the urban area increased relative to each other (p < 0.001)."
Furthermore, the young (15-24 and 25-34) are overrepresented in urban area according to the table 1, and not rural area, while those aged 45 and over are overrepresented in rural area, and not urban area.
-Also, you do not have age group 45+ according to the table 1. You can write instead: those aged 45 and over or patients aged 45 and over.

Response 5: Thank you for providing such a detailed response. We have revised the manuscript as much as possible in accordance with your suggestions. If you could review it again, we would greatly appreciate it.

Comment 6: line 11: The authors used the past tense and then the present tense. Use past tense in relation to previous text: "Across both study regions, poisoning was the most common method of self-harm, while traffic accidents are..." (WERE)....

Response 6: Please review the revised version.

Comment 7: You are not comparing traffic accidents with laceration/stabbing. Do you mean AND instead of THAN in the sentence?: "Across both study regions, poisoning was the most common method of self-harm, while traffic accidents are more common in rural regions THAN laceration/stabbing..."r

Response 7: We revised. Please review again.

-Also I suggest that you place p-values inside the sentence: …poisoning was the most common method of self-harm ($p < 0.001$), while traffic accidents are more common in rural regions ($p < 0.001$), and laceration/stabbing in urban areas ($p = 0.006$).

Response 7: A Chi-square test was performed to determine the independence of self-harm methods and study regions, and we presented one p-value for all methods of self-harm. Therefore, we revised the sentence as follows: “It was found in both study regions that poisoning was the most common method of self-harm, and that was overrepresented in rural regions. Laceration/stabbing, hanging/chocking, struck by object and fall were more overrepresented in in urban regions, but submersion, burn and traffic accident were more prevalent in rural regions ($p < 0.001$).”

Comment 8: line 13: Accoriding to the table 1, the proportion of patients in outpatient unit was higher in rural than urban regions (41 vs 37): "The proportion of patients who visited EDs through outpatient units was high in urban regions ($p < 0.001$)".

Response 3: Please accept our sincere apologies. We corrected that.

Comment 9: line 14: The word WAS is missing in the sentence. Please rephrase this sentence: A higher proportion of patients presented to EDs in an alert mental state WAS in urban regions than in rural regions from chi-square post-hoc test ($p<0.001$).

Response 9: We revised sentence as “The proportion of patients in an alert mental state who attended EDs was higher than in urban regions, whereas rural regions had a higher proportion of unresponsive patients.”.

To determine the independence of mentality distribution between two regions, a chi-square test is appropriate. Accordingly, the results of the post-hoc chi-square test have been excluded.

Comment 10: site 8, line 2: There are missing some words in this sentence: "….decreased sharply beginning the first week of February…"

Response 3: We revised as “…but decreased sharply at the first week….”

Comment 11: site 9, line 8: Change the word order in this sentence: "The proportion of self-harm patients in two age groups (15 – 24 and 25 – 34) increased significantly in 2020 ($p < 0.001$ and $p = 0.046$, respectively), whereas decreased significantly in the 35 – 44, 45 – 54, 55 – 64, 65 – 74, 75 – 84, 85 – 94 and over-95 age groups ($p <0.001$, $p = 0.003$, $p < 0.001$, $p = 0.004$, $p = 0.045$, $p <0.001$ and $p = 0.010$, respectively)."

Response 11: We revised. Please review again.

Comment 12: -site 9 line 11: Change the word ”and” to "while"in this sentence:
According to the self-harm method only the proportion of laceration/stabbing injuries increased to 29.5%,% ($p = 0.006$),AND methods such as being struck by an object, hanging/choking, falling, burningfall, submersion, burn, and traffic accidents decreased significantly ($p = 0.015$, $p < 0.001$, $p = 0.004$, $p = 0.016$, $p = 0.004$ and $p = 0.041$ respectively).

Response 12: Thank you. We revised that.

Comment 13: site 11 Table 3: In table 3 the authors calculate annual visit rate after self-harm (VRSH) by age and sex. In the method section, you only describe the weekly VRSH, but do not mention anything about the annual VRSH by age and sex.

Response 13: The related contents have been added in methods section. Please review again.

Comment 14: site 11 line 1-2: The authors wrote p=0.35. Do you mean p=0.356?" Female self-harm

visits per 100,000 people increased to 78.4 in 2020, showing a sustained increase over the previous five years (p = 0.011), whereas male self-harm visit rates did not significantly increase over time (p = 0.35) (table 3)."

Response 14: As a result of changes in statistical methods, the results and p-values have been slightly altered. Results have been updated. We request that you review this again.

Comment 15: site 11 line 2: The p-value for group aged 25-34 is <0,001 according to table 3, and not p=0.008. "There was a significant increase in standardized self-harm visit rates for groups aged 15 –24 (p = 0.003) and 25 – 34 (p = 0.008) compared to the previous five years, but a decrease for those 75 – 84 (p = 0.049), 85 – 94 (p = 0.010), 95 years and older (p = 0.011)" -Also at the end of the sentence “and” is missing before “95 years and older (p = 0.011)”.

Response 15: Thank you. We revised that.

Comment 16: site 12: The first 3 lines belongs to methods.

Response 16: Thank you. We moved the contents and revised.

Comment 17: site 12 line 9: The word WHICH is missing in this sentence: "In rural regions, the lag time between decreases in the MPMI and VRSH were 0.000 (IQR: 0.000 – 0.250) weeks, WHICH is slightly shorter than 0.500 (IQR: 0.000 -1.250) weeks seen in urban regions, but not statistically significant (p = 0.245)."

Response 17: Thank you. We corrected.

Comments from Reviewer 3

Comment 1: the authors did not respond to this comment. The response is simply a copy of one of my other original comments for the introduction. Again, please include what is the motivation for comparing rural vs urban self-harm ED usage in the introduction.

Response 1: We cited studies in East Asia which divided urban and rural regions similarly to our study, compared the effectiveness of physical distance based on regional characteristics, and noted differences in effectiveness among regions based on regional characteristics. We also indicated that the

purpose of the study is determining the differences of characteristics of self-harm patients according to region in introduction section. Please review again.

Comment 2: authors report results were corrected for multiple comparisons using the Bonferroni method and this was included in the methods. Upon review, no mention of Bonferroni correction was reported in the methods section. Please also indicate whether p-values in all the tables are corrected for multiple comparisons in the table legends.

Response 2: In response to your comments, we have modified the statistical methodology. In table 1, we conducted a chi-square test without a post-hoc test. Therefore, multiple comparisons are not necessary. Please review again.

Comment 3: In the methods, the authors write: "The Student t-test or Mann-Whitney U test was used to compare the characteristics of the two study regions (Table 1)". Which tests were t-tests/u-tests in Table. 1? Aren't the tests in Table. 1 all Chi-square tests of independence?

Response 3: Your point is absolutely correct. We used a unified compare the distribution between two groups (Chi-square tests or Fisher's exact test of independence). Thank you for your comments.

Post-hoc Chi-square tests are only done when contingency tables exceed a 2x2 design. In table 1, they do not need to be done for sex and urban/rural area. Post-hoc Chi-square tests may be done for factors with more than 2-levels, such as the age factor that has 9 levels. Post-hoc tests are typically performed using individual pairs of those levels; ie 9C2 = 36 pairs to test (ex. Ages 15-24 and 25-34 vs. sex, then ages 15-24 and 35-44 vs. sex….etc). Please clarify in the methods and results how the post-hoc Chi-square tests were done. Additionally, corrections for multiple testing will need to be done for the 36 post-hoc chi-square tests for age. Due to the high number of tests, less stringent correction methods such as Bonferroni-Holm can be considered. Here is a resource that provides some guidance on post-hoc Chi-square tests. https://alanarnholt.github.io/PDS-Bookdown2/post-hoc-tests-1.html.

Response: We are sorry for confusion. And thank you very much for your valuable feedback. After serious discussion, the authors found it unreasonable to display all 36 comparisons (9C2 = 36), because the description of the numbers did not contribute much to the study findings. Therefore, I deleted the post-hoc test results. It is possible to confirm the difference of distribution of variable between the two regions by using the Chi-square test.

Comment 4: Additionally, if more than 20% of the cells have < 5 counts for each Chi^2 test, Fisher's exact test should be used instead of Chi-square test of independence.

Response 4: Your point is very accurate, thank you. We revised the methods section. We reanalyzed the data using Fisher's exact test for "route to ED". However, the p-value was same as < 0.001.

Comment 5: where are the contingency coefficients (that the authors report they calculated) reported in table 1? They are also not mentioned in the results.

Response 5: There was no contingency coefficient in Table 1. And the statistical method was only unified with the Chi-square test, which was used to determine whether the distribution of variables between two regions was independent.

Comment 6: one-sample t-tests require a sample of observations, and then comparing the data to the expected mean. For the results in Table. 2, the authors state they are comparing the proportion (for example, of females) in 2020 to the average of the previous 5 years. The proportion of females in 2020 is a single observation, not a sample of observations, so it is not possible to do a t-test. How did the authors do t-tests for this data?

When comparing proportions, it is more appropriate to use Chi-square tests of independence. If you have an expected proportion (for example, average proportion of females from 2015 - 2019), you can perform a one-sample Chi-square test by comparing your observed proportion (ex. Proportion of females in 2020) to the expected proportion.

Response 6: You made a very valid point. However, our primary objective was to compare the values of the early pandemic era in 2020 with the trend changes of the previous five years.
One sample chi-square test is used to determine whether the distribution of the variable is the same as the expected distribution (https://www.spss-tutorials.com/spss-one-sample-chi-square-test/). Therefore, the one-sample chi-square test has the same drawback as the one-sample t-test.

Here is an example of a graph illustrating the proportion of female patients who self-harm each year. If the values in the upward trend are analyzed with a one-sample t-test, the 2020 value will appear significantly different from the average of previous years, as shown in the graph below.

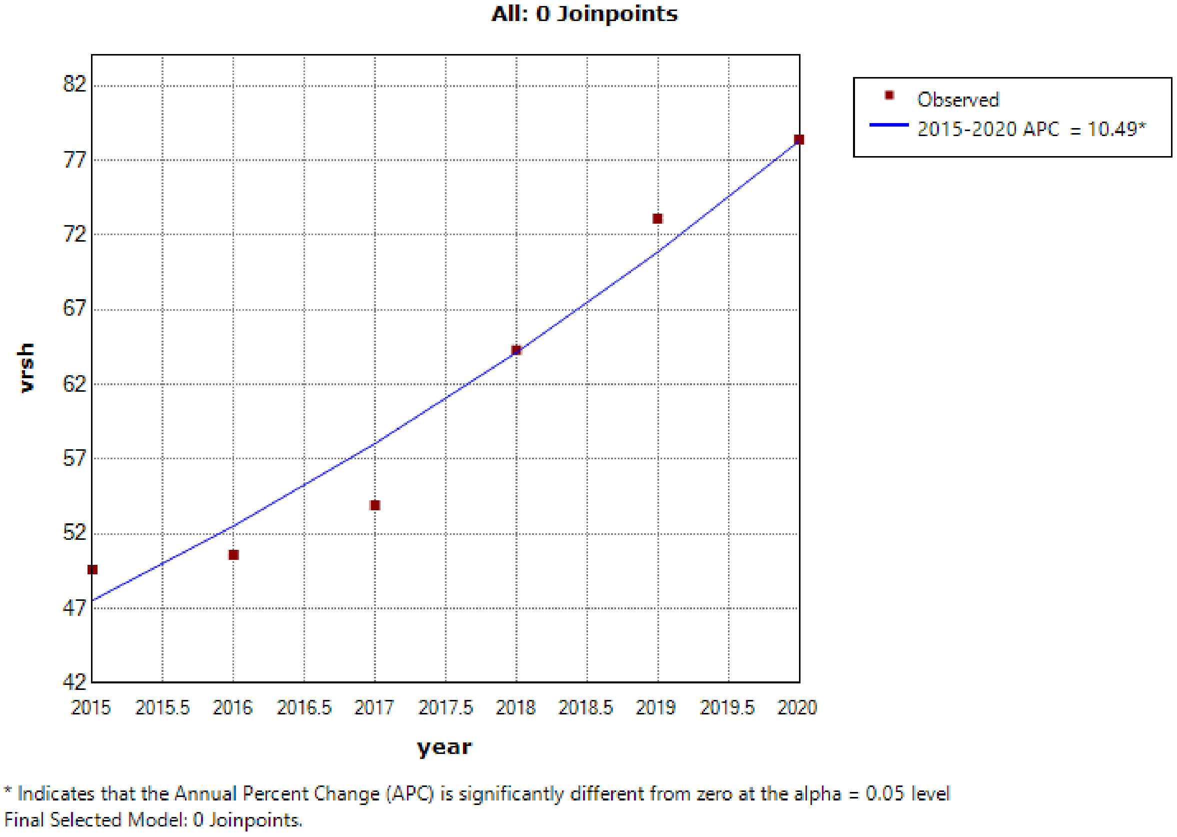

To solve this problem, we consulted to statistician and utilized a new trend analysis method.

As a result, we decided a new statistical method, joinpoint regression is employed to determine if a statistically significant change has taken place between the APC value for 2015-2019 and that of 2019-2020.

Below are the results of the joinpoint regression for the annual changes in the proportion of males among self-harm patients. From 2015 to 2019, there had been a gradual decline, followed by a sharp decline in 2020. We obtained the APC values of these two intervals and verified if there was a significant difference between the APC values of the two segments (blue versus green).

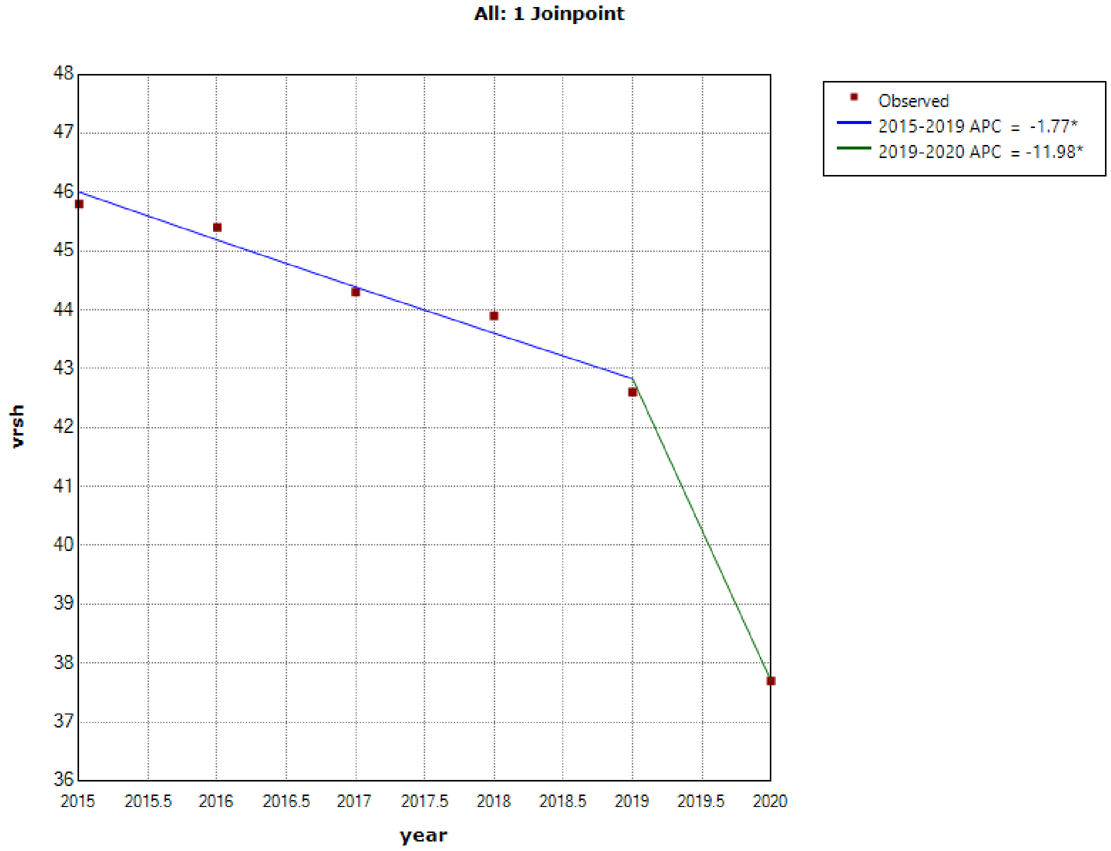

* Indicates that the Annual Percent Change (APC) is significantly different from zero at the alpha = 0.05 level
-- Test Statistic and P-Value not available for the Empirical Quantile method
Final Selected Model: 1 Joinpoint.

Through joinpoint analysis, we calculated the AAPC and APC and their confidence intervals. And the results represented in Tables 2 and 3. It would be greatly appreciated if you could review it again.

Comment 7: Table 3 legend indicates "A Shapiro-Wilk test was conducted to test for normality of the variables (2014-2018), and a one-sample t-test was performed if the normality test passed (p > 0.050)." First, the data ranges from 2015-2020, so shouldn't the authors have conducted Shapiro-Wilk tests on 2015-2019 values? Second, for one-sample t-tests, it seems the authors may have used emergency department rates from 2015-2019 as the "sample" and the emergency department rate in 2020 as the "expected mean" to compare the sample with. If this is the case, then the one-sample t-test is NOT an appropriate statistical test for this analysis because one of the main assumptions of one-sample t-tests is that all the data are independent/not-correlated. Here the data are clearly correlated since each data-point comes from the same population.

Response 7: The phrase "(2014-2018)" was mistyped as "(2015-2019)"

Comment 8: for each factor (for example, self-harm method), the authors perform a separate statistical test for each of the levels within this factor in tables 2 and 3. For example, for self-harm method, there were 11 tests performed. By chance, 5% of the results may be significant since that is what a p-value of 0.05 represents. Thus, the authors should adjust the p-values for the number of tests performed for each factor.

Response 8: According to all of review opinions, a one-sample t-test would not be appropriate to determine an increase or decrease. We re-analyzed the data using a new tool - Joinpoint regression, which is a method for comparing a change between 2019 and 2020 to a trend over the previous five years (please refer to **Response 6**)- after consulting a statistician. Although there is little change in the results, I believe it would be reasonable to revise the results. It would be greatly appreciated if you could review it again.

Other notes

"The MPMI for 2020 was significantly lower than the MPMI for 2019 based on repeated measured ANOVA analysis (p < 0.001)". Please include the F-statistic and degrees of freedom when reporting ANOVA results. Also, clarify in the methods whether it is a one, two, or three-way ANOVA since it seems two other factors were considered in figure 1: 2019/2020 and Rural/Urban. The authors imply they performed post-hoc comparisons in MPMI at specific time-points (ex. " The weekly MPMI value was higher (+2.35%) during January 2020 than during the same period in 2019 but decreased sharply beginning the first week of February (week 6 of 2020) and remained significantly lower than in 2019 until it returned to the previous year's level in the first week of April."). Please indicate in the methods how post-hoc tests were conducted and what multiple testing comparison adjustments were made.

Response:

We completely agree with your comments. A two-way ANOVA was used for the analysis. Therefore, we remarked the F and p-value obtained from the analysis in head of the results section. Additionally, all our comparison targets fell into two groups (urban versus rural, 2019 versus 2020), so an additional post-hoc analysis was not conducted.

We appreciate the advice you provided.
